# Preparation of Lignin-Based Nanoparticles with Excellent Acidic Tolerance as Stabilizer for Pickering Emulsion

**DOI:** 10.3390/polym15244643

**Published:** 2023-12-08

**Authors:** Lina Wang, Yue Kang, Weilu Zhang, Jiahao Yang, Haiming Li, Meihong Niu, Yanzhu Guo, Zhiwei Wang

**Affiliations:** 1 Liaoning Key Lab of Lignocellulose Chemistry and BioMaterials, Liaoning Collaborative Innovation Center for Lignocellulosic Biorefinery, College of Light Industry and Chemical Engineering, Dalian Polytechnic University, Dalian 116034, China; ll1713205614@163.com (L.W.); ky1249030679@163.com (Y.K.); weilu2502@163.com (W.Z.); 18931832519@163.com (J.Y.); charley1114@163.com (H.L.); niumh@dlpu.edu.cn (M.N.); 2 Guangxi Key Laboratory of Clean Pulp & Papermaking and Pollution Control, School of Light Industry and Food Engineering, Guangxi University, Nanning 530004, China; 3 Shandong Huatai Paper Co., Ltd., Dongying 275335, China

**Keywords:** lignin nanoparticles, β-alanine, Pickering emulsions, acidic tolerance

## Abstract

In this work, novel lignin-based nanoparticles (LβNPs) with high acidic tolerance were successfully prepared via electrostatic interaction between β-alanine and lignin nanoparticles. The effects of the mass ratio of lignin nanoparticles to β-alanine and pH value on the morphology and particle sizes of LβNPs were investigated with the aim of obtaining the ideal nanoparticles. The optimized LβNPs were spherical in shape with an average particle size of 41.1 ± 14.5 nm and exhibited outstanding structure stability under high acidic conditions (pH < 4). Subsequently, Pickering emulsions stabilized by LβNPs were prepared using olive oil as the oil phase. Additionally, the effects of pH value, droplet size, morphology, and storage stability on Pickering emulsions were also analyzed. The emulsions displayed excellent stability, and were stable against strongly acidic conditions (pH < 4) after 30 days of storage. The study presented a promising approach to preparing lignin-based nanoparticles with high acidic tolerance (an ideal type of stabilizer to prepare emulsions), and exhibited extremely high potential application values in the fields of drug delivery, food additives, and oily wastewater treatment.

## 1. Introduction

Pickering emulsions possess favorable properties, e.g., environmental friendliness, low cost, and higher stability against coalescence [1], which have been potentially applied in the fields of food additives [2], drug delivery [3], cosmetic manufacture [4], and other industries [5,6,7]. Generally, micro/nanoparticles are usually employed as the stabilizers for Pickering emulsions, which are easily adsorbed onto the oil–water interface, prevent the adhesion of emulsion particles to form unstable large aggregates, and provide kinetic stability for emulsions [8]. The applied micro/nanoparticles include, but are not limited to, ZnO nanoparticles [9], polyurethane-based nanoparticles [10], Fe_3_O_4_ nanoparticles [11], and poly(lactic acid) microparticles [12]. Among the above micro/nanoparticles, inorganic and synthetic polymer particles typically exhibit low biocompatibility and poor degradability [13], and thus cause some safety issues in the resulting emulsions. In the past decades, extensive literature has reported the possibility of particles from biopolymers, e.g., lignin [14], chitosan [15], protein [16], and polysaccharide [17], stabilizing Pickering emulsions.

As a kind of aromatic biopolymer, lignin has a hydrophilic hydroxyl group and hydrophobic benzene ring structure, and it has been employed in various applications such as emulsion stabilizers due to its amphiphilic property [18,19]. Currently, various lignin-based particles with different sizes, e.g., microparticles (1 μm~100 μm) [20], sub-microparticles (100 nm~1.0 μm) [21], and nanoparticles (1 nm~100 nm) [22,23], have been developed as effective stabilizers for Pickering emulsions. Compared to colloidally unstable and interpenetrating micro- and larger-sized lignin particles, the lignin nanoparticles (LNPs) display enhanced adsorption ability at the oil–water interface of the emulsions, which are beneficial to the formation of stable and homogeneous Pickering emulsions [24]. Up to now, various methods have been explored to prepare LNPs, e.g., acidic precipitation [25], solvent exchange [26], solvent–antisolvent [27], ultrasonication polymerization [28], biological treatment [29], etc. The solvent–antisolvent technology is the most commonly used method due to its easy implementation process and the generated LNPs with controllable sizes. In brief, lignin is initially dissolved in some organic solvents, e.g., acetone [30], dimethyl sulfoxide [31], tetrahydrofuran [32], and methanol [33], and then excess anti-solvents, e.g., water, are injected into the raw lignin solution to induce the self-assembly behavior of lignin through the hydrophobic effect. However, the previously reported organic solvents are usually toxic and with poor biocompatibility, while parts of these solvents exhibit limited ability to dissolve lignin. Some green solvents, e.g., ethanol, have been used for the preparation of LNPs; however, the low yields prevented easy large-scale preparation [34]. Therefore, the first objective of this study was to find a green and inexpensive solvent to the lignin and prepare the LNPs with controllable sizes through the solvent–antisolvent method.

Up to now, the LNPs have been extensively involved in the preparation of Pickering emulsions as stabilizers. The reported stabilized Pickering emulsions are usually stable neutral [35] or weak acid conditions (4 < pH < 7) [36], which are often demulsified in highly acidic conditions due to the decreased electrical charges on the surface of LNPs [37,38]. However, in some applying conditions of Pickering emulsions, e.g., the separation of oily wastewater in highly acidic environments, the pH values are lower than 4.0 [39]. Therefore, there is a need to develop some approaches to achieve the Pickering emulsions with high acidic tolerance. Sipponen et al. [40] have prepared cationic colloidal lignin particles by adsorbed cationic lignin onto LNPs, which exhibit excellent emulsion stability in the conditions with pH value from 2 to 6. Pang et al. [41] have obtained lignin/sodium dodecyl sulfate (SDS) composite nanoparticles (LSNPs) via acidic precipitation, which exhibited excellent emulsifying ability in conditions with pH values from 2.5 to 11.0. However, the generated emulsions can be only stable for 10 min, which is not beneficial to their practical application. Therefore, the second aim of this manuscript is to prepare lignin-based nanoparticles as stabilizers for Pickering emulsions, with excellent stability in highly acidic conditions.

In this paper, the lignin-based nanoparticles (LNPs) were initially prepared via the solvent–antisolvent method, in which the green solvent of γ-valerolactone (GVL) was used to dissolve lignin instead of other toxic organic solvents. Subsequently, the LNPs were combined with β-Alanine (β-Ala), a green type of organic surfactant [42], to prepare the LNPs/β-Ala complex nanoparticles (LβNPs) with the aim of improving the high acidic tolerance of LNPs. The morphology, particle size, and zeta potential of the LNPs and LβNPs were characterized and the effects of the preparation conditions on the sizes of LNPs and LβNPs were evaluated. Finally, the LβNPs were used as the stabilizers and olive oil as the oil phase to prepare Pickering emulsions, and their sizes, morphologies, and high acidic tolerance were observed by confocal laser scanning microscopy (CLSM) and optical microscope. The findings in this manuscript could provide highlights for preparing lignin-based nanoparticles as the Pickering emulsions stabilizers with high acidic tolerance instead of conventional stabilizers.

## 2. Materials and Methods

### 2.1. Materials

Kraft lignin (KL) was obtained from FPInnovations (Thunder Bay, Canada). Sodium hydroxide (AR grade), tetrahydrofuran (THF, AR grade), sodium chloride (AR grade), and hydrochloric acid (AR grade) were purchased from Tianjin Kemi O Chemical Reagent Co., Ltd. (Tianjin, China). β-Alanine (β-Ala, 99%) was purchased from Macklin Biochemical Co., Ltd. (Shanghai, China). γ-Valerolactone (GVL, AR grade) was purchased from Aladdin Biochemical Technology Co., Ltd. (Shanghai, China). All of the chemicals were directly used without further purification.

### 2.2. Preparation of LNPs

LNPs were prepared by the solvent–antisolvent method [43]. Briefly, KL (5 mg) was sufficiently dissolved into 10 mL of GVL/water solutions (GVL: water = 0:10–10:0, v:v) under magnetic stirring at 700 rpm. Subsequently, 40 mL of deionized water was continuously injected into the GVL/water solutions under magnetic stirring. Typically, the initial concentration of LNPs in prepared suspension was around 1 g/L. After 12 h magnetic stirring, the suspension was further transferred into a dialysis bag (Igbo Bio, 8000–14,000 Da) and dialyzed against deionized water for 24 h. The obtained LNPs suspensions were stored at room temperature until use.

### 2.3. Preparation of LβNPs

In brief, 1 mL, 2 mL, 3 mL, 5 mL, 7 mL, and 10 mL of β-Ala solution with a concentration of 1.0 g/L were added into 10 mL of the above prepared LNPs suspension (1 g/L) under magnetic stirring at 700 rpm. The mass ratio of β-Ala to LNPs ranged over 0.1:1.0–1.0:1.0. The mixture suspension was then shaken on a rotary shaker at 200 r/min for 6 h at 25 °C. Suspensions with different mass ratios of β-Ala to LNPs (0.1:1.0–1.0:1.0, *w/w*) were obtained, which were dialyzed against deionized water in dialysis membrane (MWCO 8000–14,000 Da) for 5 days. The pH values of final LβNPs suspensions were adjusted to 3.0 with 20 wt.% HCl and NaOH.

### 2.4. Preparation of LβNPs Stabilized Pickering Emulsions

To prepare Pickering emulsions, the freshly fabricated LβNPs suspensions (0.1 wt.%, 0.2 wt.%, and 0.4 wt.%) were mixed with olive oil with different oil–water ratios (1:9–3:7, *v/v*). Subsequently, 10 mL of mixture was emulsified by sonicating at 180 W for 1 min with 2 s working time for 3 s break time to obtain Pickering emulsion. The emulsions were stored in glass bottles for 30 days at room temperature for stability evaluation. 

### 2.5. Characterization of LNPs, LβNPs, and Pickering Emulsions

The diameter, polydispersity index (PDI), and zeta potential of all samples were determined by Nano-ZS ZEN90 (Malvern Instruments Ltd., Worcestershire, UK), and the values were collected by three consecutive measurements. A backscattering mode (detection angle = 175°) was adopted and the particle size distribution was expressed on the basis of number frequency. Results are presented graphically by Origin 2021 software, with the error bars reflecting the results from triplicate experiments. TEM images of LNPs and LβNPs were recorded with transmission electron microscopy (H-7650, Hitachi, Tokyo, Japan) at an accelerating voltage of 200 kV. One drop of the LβNPs and LNPs suspension (1 mg/mL) was injected into copper grids with a carbon film and then dried at room temperature. The Fourier transform infrared (FT-IR) spectra of KL and LNPs were conducted to characterize the chemical structure on a PerkinElmer Fourier I spectrometer (Waltham, MA, America). The UV–vis spectra of LNPs were characterized by a UV-vis spectrophotometer (UH-5300, Hitachi, Tokyo, Japen). The morphologies of LβNPs-stabilized Pickering emulsions were analyzed on an optical microscope (DM-2700P, Leica, Wetzlar, Germany). The morphology of emulsions was analyzed at 40× magnification using a confocal laser scanning microscope (CLSM, Leica TCS-SP5, Leica, Wetzlar, Germany). Approximately 10 μL of Nile Red (1 mg/mL in dimethyl sulfoxide) was used to stain the oil phase (argon laser with an excitation line at 488 nm).

### 2.6. Stability Measurement of Pickering Emulsions

The effects of pH on the particle size distribution and surface charge of the emulsions were examined by adjusting pH to the desired values (1.0–8.0) using 1 M NaOH or HCl. The storage stability of LβNPs-stabilized Pickering emulsions was tested. The emulsions were packed in glass bottles and stored at room temperature (25 ± 2 °C) for 30 days. The change in droplet sizes was observed by optical microscopes.

## 3. Results and Discussion

### 3.1. Preparation and Characterization of LNPs

In this work, the LNPs were prepared from kraft lignin (KL), a type of industrial lignin with high annual output, using the solvent–antisolvent method. The green solvent of GVL was employed as the solvent and combined with water to dissolve kraft lignin. We have also compared the effects of different solvents on the particle size, zeta potential, and PDI of LNPs (Table S1). As is shown in Table S1, the LNPs with GVL as the solvent had a higher negative zeta potential and lower PDI than those prepared by ethanol. Moreover, the LNPs prepared by ethanol exhibited a smaller average particle size (52.51 nm) and relatively low yield (36%). Therefore, GVL was selected as the dispersive solvent for the formation of the LNPs.

**Figure 1:**
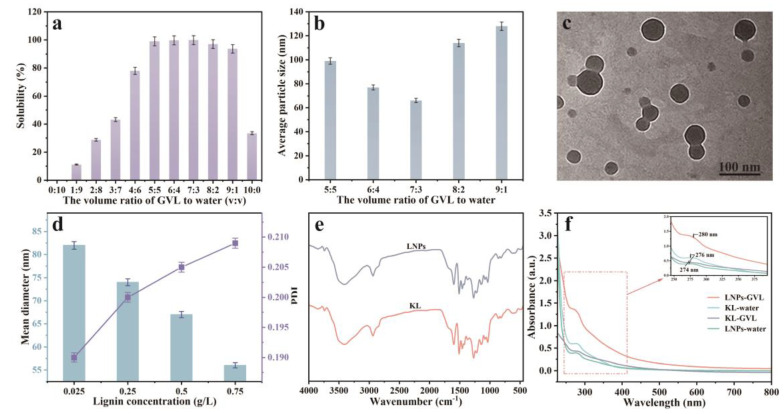
(**a**) Solubilities of KL in the GVL/water mixtures, (**b**) average sizes of LNPs prepared with various volume ratios of GVL to water, (**c**) TEM image, and (**d**) mean diameter and PDI of LNPs prepared with 7:3 (v:v) volume ratio of GVL to water. (**e**) FT-IR, and (**f**) UV-vis absorption spectra of KL and LNPs samples.

In order to obtain the LNPs with ideal sizes, the effects of the volume ratio of GVL to water on the solubility of KL and the average sizes of prepared LNPs were evaluated. The results are shown in Figure 1. As shown in Figure 1a, the solubility of KL was increased from 0.12% to 99.88% with increasing the volume ratio of GVL to water from 0:10 to 7:3, and was decreased from 99.88% to 33.49% when the volume ratio of GVL to water was increased from 7:3 to 10:0. The maximum solubility of lignin (99.88%) was obtained with a 7:3 volume ratio of GVL to water. This could be explained by Hildebrand solubility theory [44]; the maximum solubility of lignin should be obtained when the solubility parameter of the solvent mixture is close to the solubility parameters of lignin. The effects of the volume ratio of GVL to water on the particle sizes of LNPs were investigated and the results are shown in Figure 1b. The average particle size of LNPs was decreased from 99 nm to 66 nm with increasing the volume ratio of GVL to water from 5:5 to 3:7, and was increased from 66 to 128 nm with further enhancing the volume ratio of GVL to water from 3:7 to 9:1. The minimum average particle size of LNPs (66 nm) was achieved with a 7:3 volume ratio of GVL to water.

As shown in Figure 1c and Figure S2, the LNPs displayed a uniform spherical shape and well-dispersed ability in aqueous solution. The average size of the LNPs by nano-size analysis was 66 nm. The LNPs prepared with a 7:3 volume ratio of GVL to water were selected for the subsequent experiments. As shown in Figure 1d, the mean diameter of LNPs was decreased from 82 nm to 56 nm with lignin concentration increasing from 0.025 g/L to 0.75 g/L. The PDI was increased from 0.190 to 0.209 with increasing lignin concentration as the degree of particles’ agglomeration was enhanced at higher concentrations.

The FT-IR spectra of KL and LNPs are shown in Figure 1e. The FT-IR spectra of KL and LNPs displayed typical absorption peaks at 1600, 1510, and 1459 cm^−1^, which correspond to the aromatic skeletal vibrations of lignin. Additionally, the peak at 1685 cm^−1^, attributed to conjugated carbonyl stretching vibration, was observed. Additionally, the phenolic and aliphatic hydroxyl groups in lignin were confirmed by a broad absorption band at 3100–3600 cm^−1^. Overall, no significant changes were observed between the lignin and LNPs. The UV-vis absorption spectra of the KL and LNPs are shown in Figure 1f. The absorbance peaks at ~280 nm which correlated to phenylpropanoid units of lignin were present in all studied lignin samples. Both the UV spectra of KL in water and in GVL exhibited strong absorption peaks at 276 nm, indicating that the solvents had no effect on the absorption peak. The LNPs in GVL exhibited an absorption peak at 274 nm, which was red-shifted compared to the absorption peak at 280 nm of the LNPs in water. The results could be attributed to the π-π interactions in the preparation progress of LNPs between lignin molecules.

### 3.2. Preparation and Characterization of LβNPs

**Figure 2:**
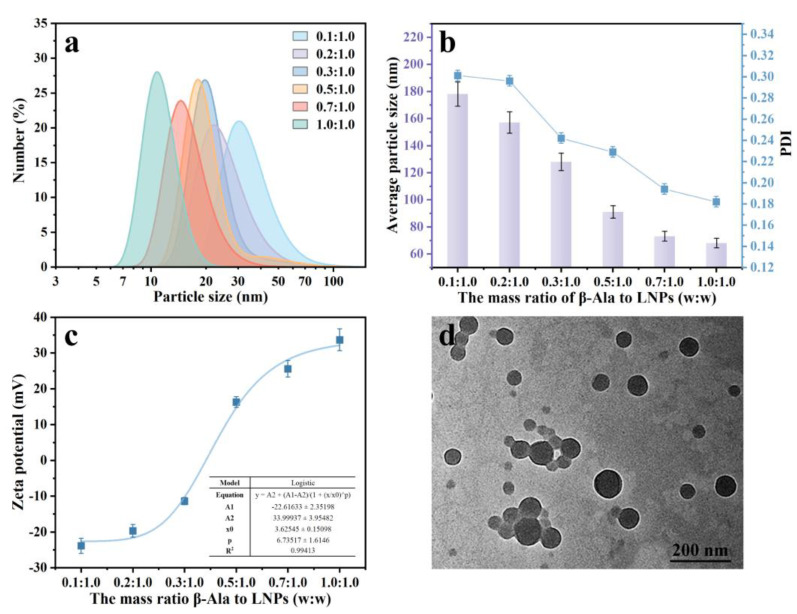
(**a**) Particle size distribution, (**b**) average particle sizes, PDI, and (**c**) zeta potentials of LβNPs prepared with different mass ratios of β-Ala to LNPs, (**d**) TEM image of LβNPs prepared with 1.0:1.0 (w:w) mass ratio of β-Ala to LNPs.

We found that the prepared LNPs were easily aggregated and precipitated when the pH value was lower than 4.0 (Figure S1), which was probably due to the protonation of carboxyl groups and thus led to the disappearance of electrostatic repulsion between LNPs. As a type of green organic surfactant, β-Ala was composited with LNPs to prepare lignin-based nanoparticles (LβNPs) with high acidic tolerance through the electrostatic interaction between LNPs and β-Ala. The selected β-Ala would provide a positive amino group for the LβNPs. The effect of the mass ratio of β-Ala to LNPs on morphology, size, and zeta potential of the LβNPs was evaluated and the results are shown in Figure 2. During this experiment, the pH value of LβNPs solution was constant at 3.0. As shown in Figure 2a, the particle size distribution of LβNPs shifted from larger size to smaller sizes with increasing the mass ratio of β-Ala to LNPs from 0.1:1.0 to 1.0:1.0. With increasing the mass ratio of β-Ala to LNPs from 0.1:1.0 to 1.0:1.0, the particle sizes of LβNPs were decreased from 47.6 ± 20.4 nm to 10.79 ± 14.5 nm (Figure 2b). The PDI of LβNPs decreased from 0.301 to 0.182 with increasing mass ratio of β-Ala to LNPs (Figure 2b). All the PDI values of LβNPs were less than 0.4, indicating a moderately homogeneous size distribution.

The effect of the mass ratio of β-Ala to LNPs on the zeta potential of LβNPs is shown in Figure 2c. The R2 of the experimental data was 0.994, indicating that the curve in Figure 2c was best described by the Logistic model. However, their zeta potentials were increased from −23.9 mV to +33.7 mV (Figure 2c). Interestingly, the zeta potential was conversing from negative (−11.4 mV) to positive (+6.3 mV) when the mass ratio of β-Ala to LNPs was elevated from 0.3:1.0 to 0.5:1.0, suggesting that the negative LNPs had even been neutralized by positive β-Ala, and resulting in positive LβNPs. As shown in Figure 2d, the LβNPs prepared with 1.0:1.0 mass ratio of β-Ala to LNPs displayed a spherical shape and were well mono-dispersed, with average particle size determined to be 41.1 ± 14.5 nm by ImageJ software (version 2.0.0); these were selected for the subsequent experiments.

**Figure 3:**
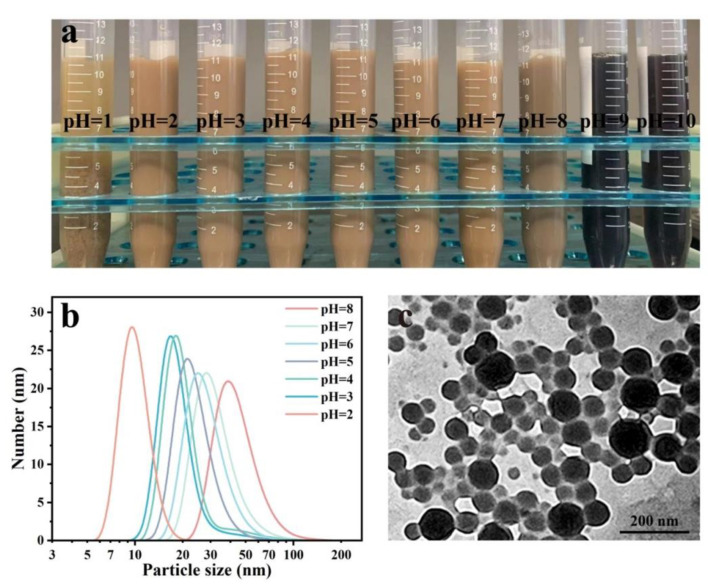
(**a**) Image of LβNPs suspension with different pH values, (**b**) particle size distribution of LβNPs with different pH values, (**c**) TEM image of LβNPs with a pH value of 3.0.

The effect of pH value on morphology, zeta potential, and particle distribution of LβNPs was analyzed (Figure 3). The LβNPs were aggregated and precipitated in the water with a pH value of 1, and were stable with the pH value in the range of 2–7. With further increasing the pH values to alkaline values in the range of 8–10, the LβNPs were dissolved into the water.

As observed in Figure 3b, the LβNPs in the solution with a pH value of 2 had the smallest particle and narrowest size distribution; sizes were ranged from 5 nm to 30 nm. As shown in Figure 3c, the LβNPs displayed spherical nanoparticles with an average size of 55.30 ± 16.67 nm and well-dispersed ability in aqueous solution. These results demonstrated that the as-prepared LβNPs remained stable in a highly acidic solution.

As is shown in Figure S4, the storage stabilities of the LβNPs and LNPs were assessed considering their changes in zeta potential during various storage times (0–30 days) at 25 °C. After storage for 30 days, the zeta potentials of LNPs and LβNPs were decreased from −39.3 mV to −29.9 mV and from +42.6 mV to +33.4 mV, respectively. At the same time, the absolute value of the zeta potential of LβNPs was higher than that of LNPs, indicating their better storage stability than LNPs. In addition, the absolute values of the zeta potentials were higher than 30 mV, indicating the LβNPs and LNPs exhibited excellent stability.

### 3.3. Preparation and Characterization of LβNPs-Stabilized Pickering Emulsions

**Figure 4:**
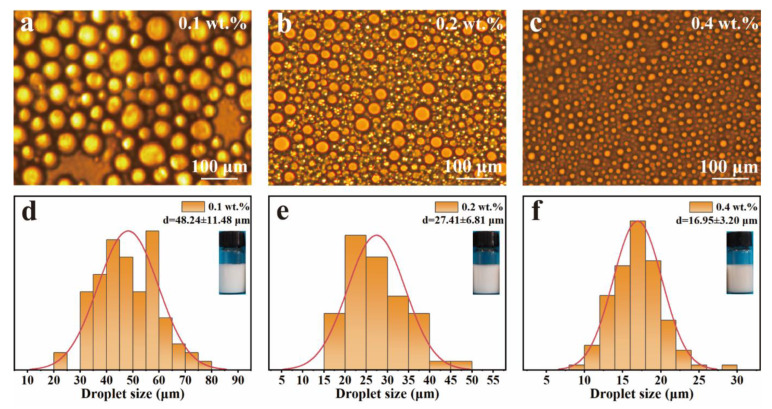
Optical micrographic images of Pickering emulsions with (**a**) 0.1 wt.%, (**b**) 0.2 wt.%, (**c**) 0.4 wt.% mass fraction of LβNPs (in versus of emulsions mass), and (**d**–**f**) their droplet size distribution (**d**: 0.1 wt.%, **e**: 0.2 wt.%, **f**: 0.4 wt.%).

The Pickering emulsions were successfully prepared by ultrasonic treatment with the LβNPs as stabilizers and the olive oil as the oil phase. The droplet sizes and morphologies of LβNPs-stabilized Pickering emulsions were analyzed by optical microscope. The effect of lignin concentration on morphology and stabilization of Pickering emulsions was explored and the results are found in Figure 4. As shown in Figure 4a–c, the droplets of emulsions were spherical in shape and well distributed. As observed in Figure S4, all of the prepared Pickering emulsions were stable against oiling-off. The particle sizes of emulsions were analyzed using ImageJ software (version 2.0.0), taking at least 100 individual droplets for evaluation. The results are shown in Figure 4d–f. The droplet size was decreased from 48.24 ± 11.48 μm to 16.95 ± 3.20 μm with increasing mass ratio of LβNPs (in versus of emulsions mass) from 0.1 wt.% to 0.4 wt.%.

**Figure 5:**
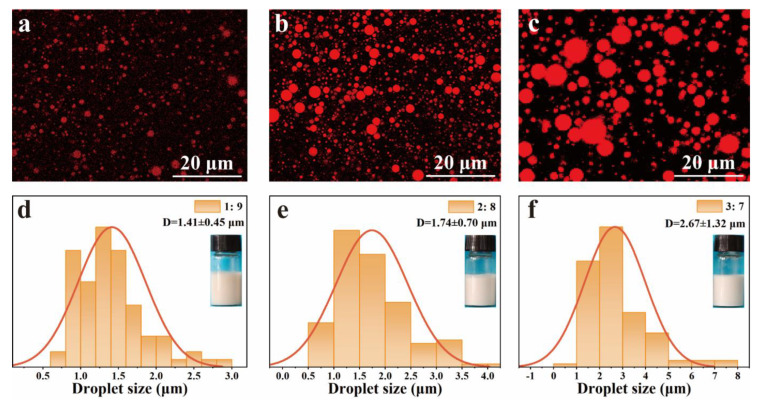
Confocal laser scanning microscopy (CLSM) images of emulsions with (**a**) 1:9, (**b**) 2:8, (**c**) 3:7 volume ratio of oil to water, and their droplet size distributions (**d**): 1:9, (**e**): 2:8, (**f**): 3:7.

The effect of the volume ratio of oil to water on the emulsion droplet size and morphology of the Pickering emulsions was investigated and the results are shown in Figure 5. The oil phase (olive oil) was stained with Nile red and observed using the CLSM. As shown in Figure 5a–c, the emulsion droplet sizes were obviously increased from 1.41 ± 0.45 μm to 2.67 ± 1.32 μm with increasing the volume ratio of oil to water from 1:9 to 3:7.

**Figure 6:**
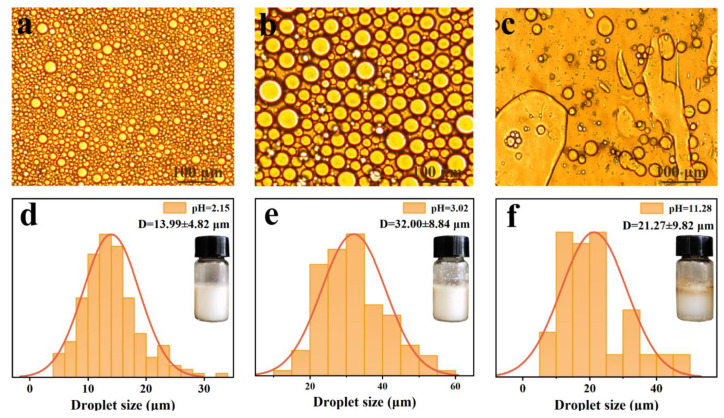
Optical micrographic images of emulsions with pH value of: (**a**) 2.15, (**b**) 3.02, (**c**) 11.28; and their droplets size distributions (**d**): 2.15, (**e**): 3.02, (**f**): 11.28.

In order to obtain Pickering emulsions with high acidic tolerance, the effect of pH value on their morphology and stability is shown in Figure 6. As depicted in Figure 6a, b, the LβNPs-stabilized Pickering emulsions were homogeneously spherical in shape, showed uniform emulsion droplet distribution in an acidic medium, and the droplet sizes of the emulsions were in the range of 5–60 μm. With elevating the pH value to 11, the oil droplets aggregated into non-spherical droplets due to the dissolution of lignin in the alkaline condition, and could not stabilize the interface of oil–water.

The average droplet sizes of the emulsions with a pH value of 2.15 and 3.02 were 13.99 ± 4.82 μm and 32.00 ± 8.84 μm, respectively, both of which were lower than that with a pH value of 11.28. The results verified that LβNPs were thermodynamically stable in highly acidic conditions due to electrostatic interaction between the β-Ala and LNPs, which would prevent the coalescence of emulsions.

### 3.4. pH-Responsive Behavior of Pickering Emulsions

**Figure 7:**
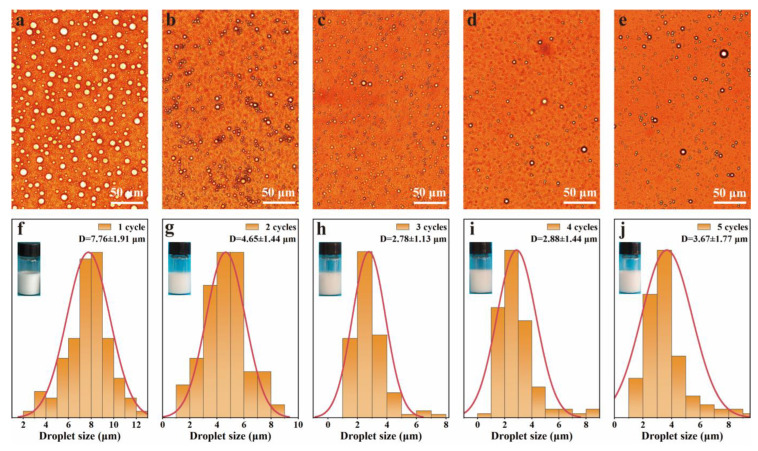
pH-Responsive performance of the emulsions. (**a**–**e**) optical images and (**f**–**j**) droplets size distribution of emulsions at pH 3.0 after 1 to 5 cycles of switching the pH value between 3.0 and 11.0.

In order to obtain a stable emulsion, the repeatability of emulsions was investigated by a pH-responsive cycling experiment. The optical microscope images and droplet size distributions of emulsions after five cycles are shown in Figure 7. The droplets exhibited spherical shape, and uniform droplet dispersion with sizes of 2–8 μm (Figure 7a–e). The droplet size distribution and photos of the emulsions are shown in Figure 7f–j. The emulsions were still stable and without demulsification for at least five cycles. The type and droplet size of the regenerated emulsions were almost the same as those of the original emulsions. The droplet sizes of emulsions decreased from 7.76 ± 1.91 μm to 2.78 ± 1.13 μm after one to three cycles, which slightly increased with the increase in cycles from three to five. These results demonstrated that the emulsion had outstanding stability against the changes in pH value.

### 3.5. Stability of Pickering Emulsions during Storage

**Figure 8:**
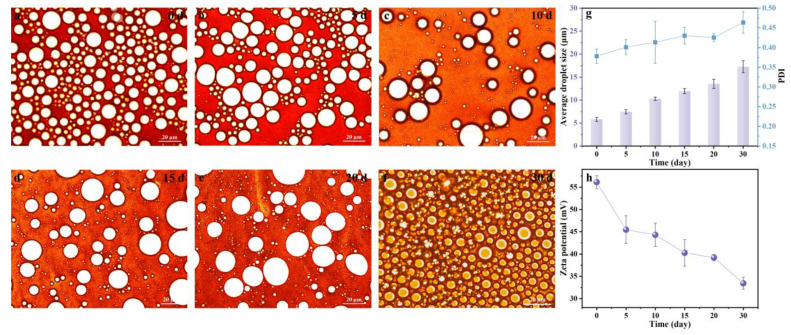
Effect of storage time on (**a**–**f**) morphology, (**g**) average droplet size, PDI, and (**h**) zeta potential of Pickering emulsions.

To evaluate a realistic application of the LβNPs Pickering emulsions, the long-term storage stability of Pickering emulsions was monitored for 30 days at pH 3.0 (Figure 8). The morphologies of the fabricated emulsions were observed for 30 days by optical microscopy. As shown in Figure 8a–f, all the droplets exhibited homogeneously spherical shapes. The average droplet sizes of the emulsions were slightly increased from 5.82 μm to 17.25 μm with increasing storage time from 0 day to 30 day. The PDI of the emulsions was increased from 0.378 to 0.464, maintaining a value lower than 0.5. Moreover, the absolute zeta potential of the emulsions (Figure 8h) was higher than 30 mV, indicating that all of the emulsions possessed excellent storage stability in highly acidic conditions.

## 4. Conclusions

In this study, a novel strategy was proposed to prepare lignin-based nanoparticles (LβNPs) through the electrostatic interactions between lignin nanoparticles and β-Alanine. The obtained LβNPs exhibited a spherical shape and uniform particle size, and were well dispersed with an average diameter of 41.1 ± 14.5 nm. Subsequently, the LβNPs were adopted as stabilizers to prepare Pickering emulsions with olive oil as the oil phase. The droplet sizes of emulsions (pH = 3.0) were 13.99 ± 4.82 μm prepared with a 3:7 volume ratio of oil to water. Furthermore, the LβNPs-based Pickering emulsions prompted a long-term storage stability (30 days) against coalescence in highly acidic conditions (pH = 3.0). Overall, this work offers a facile and green strategy for the valorization of kraft lignin in high-valued applications, e.g., drug delivery, food additive, and oily wastewater treatment fields.

## Data Availability

Data are contained within the article and Supplementary Materials.

## Supplementary Materials

The following supporting information can be downloaded at: https://www.mdpi.com/article/10.3390/polym15244643/s1, Figure S1: The effect of pH value on the status of LNPs suspension, arranged in order of pH 1.0 (left) to pH 12.0 (right); Figure S2: The particle size distribution of LNPs suspension with 7: 3 volume ratios of GVL to water; Table S1: The average particle size, zeta potential, PDI, and yield of LNPs from the process dissolved in ethanol and those using GVL as a solvent; Figure S3: TEM images of LβNPs prepared with different mass ratios of β-Ala to LNPs: (a) 1.0:1.0; (b) 0.7:1.0; (c) 0.5:1.0; (d) 0.3:1.0; (e) 0.2:1.0; (f) 0.1:1.0; Table S2: The average particle size, zeta potential, and PDI of LβNPs at pH 6.7 and pH 3.0; Figure S4: Effect of storage time on the average particle size, zeta potential, and PDI of LNPs and LβNPs; Figure S5: Effect of the concentration of lignin (wt.%) on status of Pickering emulsion; Figure S6: Effect of the mass ratio of β-Ala to LNPs on status of Pickering emulsion; Figure S7: Effect of the pH value on status of Pickering emulsion.
