# Supplementary material for: Preparation of Lignin-Based Nanoparticles with Excellent Acidic Tolerance as Stabilizer for Pickering Emulsion
Source: Polymers (Basel). 2023 Dec 8;15(24):4643. doi: 10.3390/polym15244643 (PMC10747945; doi:10.3390/polym15244643)
Supplement: Supplementary file 1 [file polymers-15-04643-s001.zip › polymers-2689627-supplementary.pdf]

Supplementary Information

Preparation of lignin-based nanoparticles with excellent acidic tolerance as stabilizer for Pickering emulsion

*Lina Wang a, 1, Yue Kang a, 1, Weilu Zhang a, Jiahao Yang a, Haiming Li a, Meihong Niu a, Yanzhu Guo a, b, c, * and Zhiwei Wang b, **

a Liaoning Key Lab of Lignocellulose Chemistry and BioMaterials, Liaoning Collaborative Innovation Center for Lignocellulosic Biorefinery, College of Light Industry and Chemical Engineering, Dalian Polytechnic University, Dalian, 116034, China;

b Guangxi Key Laboratory of Clean Pulp & Papermaking and Pollution Control, School of Light Industry and Food Engineering, Guangxi University, Nanning, 530004, China;

c Shandong Huatai Paper Co., Ltd., Dongying, 275335, China.

1 Lina Wang and Yue Kang contributed equally to this work.

** Corresponding Authors E-mail: guoyz@dlpu.edu.cn (Yanzhu Guo), wangzhiwei@gxu.edu.cn (Zhiwei Wang); Tel.: +86-15164033963 (Yanzhu Guo)*

Preparation and characterization of LNPs

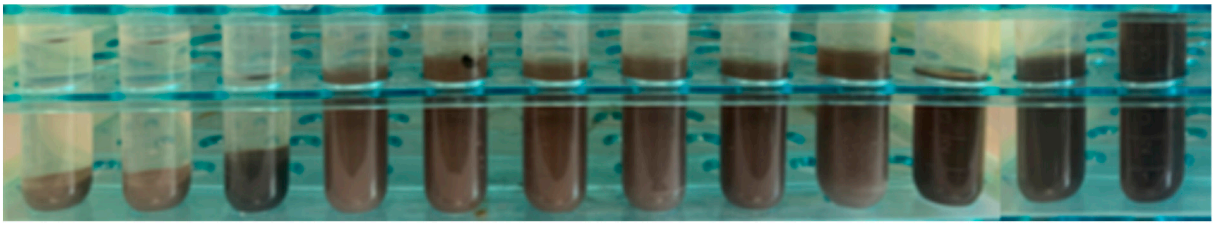

Figure S1. The effect of pH value on the status of LNPs suspension, that are arranged in order of pH 1.0 (left) to pH 12.0 (right).

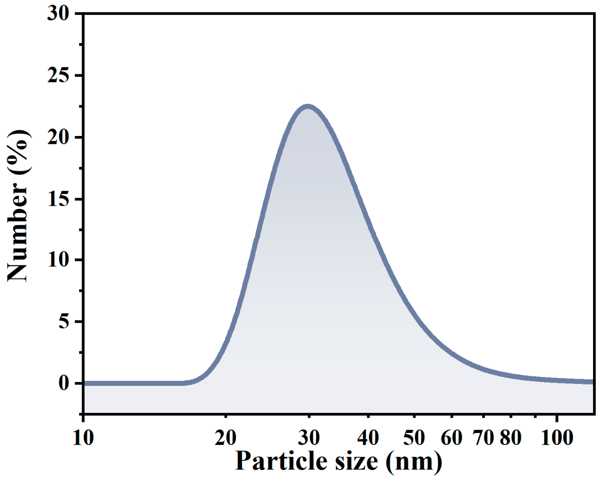

Figure S2. The particle size distribution of LNPs suspension with the 7: 3 volume ratios of GVL to water.

As is shown in Table S1, the LNPs which GVL as the solvent had a higher negative zeta potential and lower PDI compared to the LNPs prepared by ethanol. However, the LNPs prepared by ethanol exhibited smaller average particle size and with relatively small yield value. In conclusion, on the basis of higher percentage yield, better zeta potential as well as PDI, the GVL was selected as the solvent for the formation of the LNPs.

Table S1. The average particle size, zeta potential, PDI, and yield of LNPs from the process dissolved in ethanol and those using GVL as a solvent.

Solvents	Average particle size (nm)	Zeta potential (mV)	PDI	Yield (%)
GVL	94.20	-33.3	0.113	76
Ethanol	52.51	-29.2	0.666	36

Preparation and characterization of LβNPs

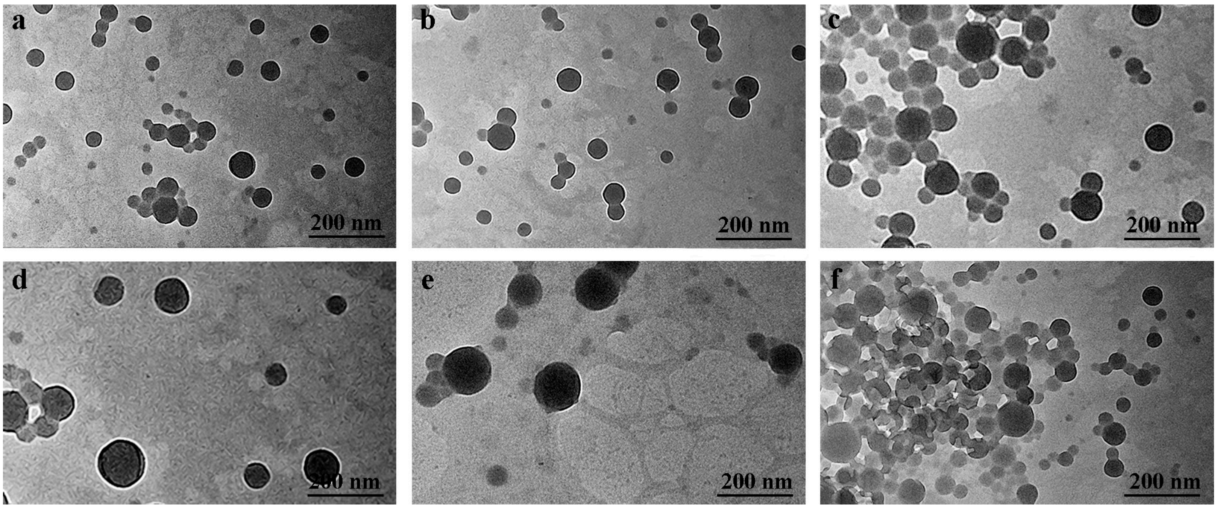

Figure S3. TEM images of LβNPs prepared with different mass ratios of β-Ala to LNPs: (a) 1.0:1.0; (b) 0.7:1.0; (c) 0.5:1.0; (d) 0.3:1.0; (e) 0.2:1.0; (f) 0.1:1.0.

As shown in Table S2, the average particle size, zeta potential, and PDI of LβNPs at various initial pH. The LβNPs at pH 3.0 showed smaller average particle size as well as PDI, and higher positive zeta potential compare with those at pH 6.7. Therefore, pH 3.0 was selected as the best operational pH value.

Table S2. The average particle size, zeta potential, and PDI of LβNPs at pH 6.7 and pH 3.0.

	Average particle size (nm)	Zeta potential (mV)	PDI
LβNPs (pH 6.7)	90.2	+36.9	0.297
LβNPs (pH 3.0)	64.1	+37.2	0.290

The zeta potential of LNPs was decreased from -39.3 mV to -29.9 mV with time, whereas the zeta potential of LβNPs was decreased from +42.6 mV to +33.4 mV. At the same time, the absolute value of zeta potential of LβNPs was higher than that of LNPs according to the above zeta potential measurements, indicating their better storage stability than LNPs.

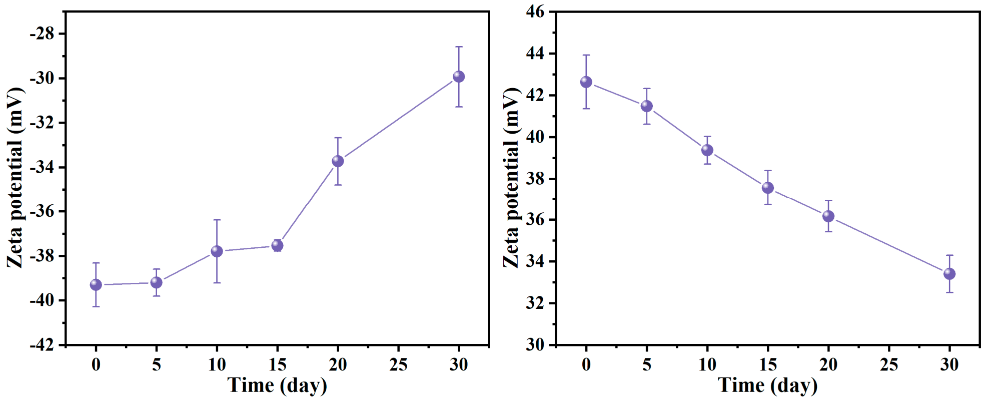

Figure S4. Effect of storage time on the average particle size, zeta potential, and PDI of LNPs and LβNPs.

Preparation and characterization of LβNPs stabilized Pickering emulsions.

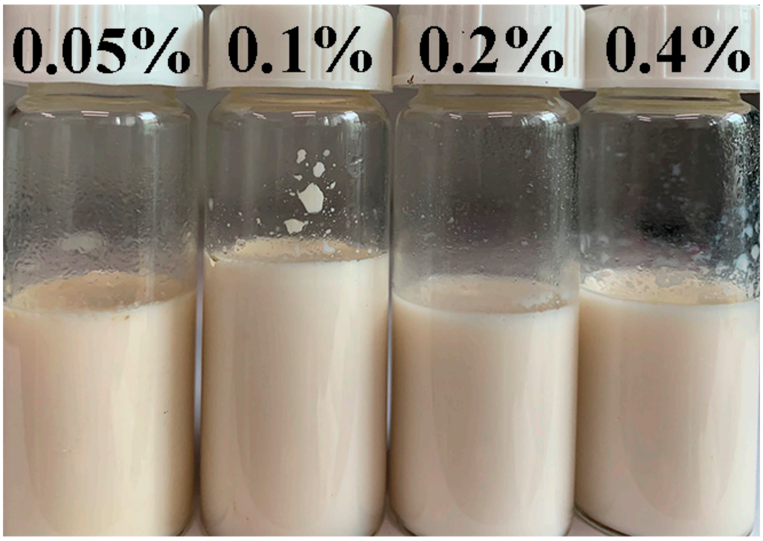

Figure S5. Effect of the concentration of lignin (wt.%) on status of Pickering emulsion.

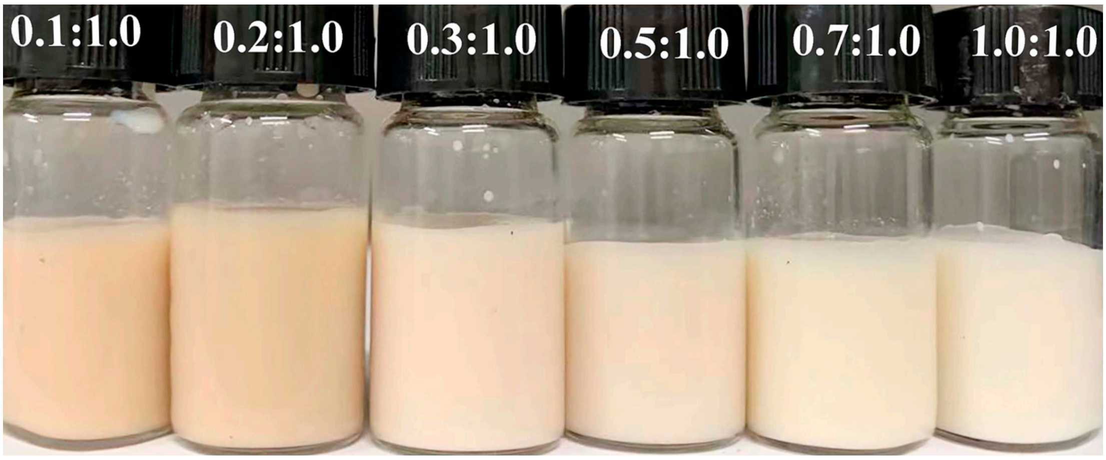

Figure S6. Effect of the mass ratio of β-Ala to LNPs on status of Pickering emulsion.

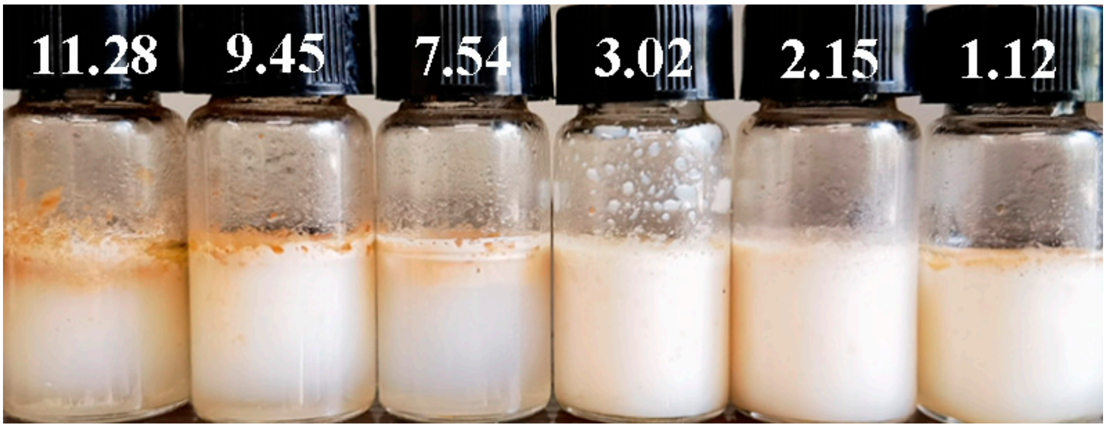

Figure S7. Effect of the pH value on status of Pickering emulsion.
